# Immunoexpressions of PD-L1 and EZH2 in Endometrial Carcinoma: Associations with Clinicopathological Parameters

**DOI:** 10.3390/diagnostics15081042

**Published:** 2025-04-19

**Authors:** Badrul Iskandar Abdul Wahab, Shamsul Azhar Shah, Roslina Mohd Arshad, Nurwardah Alfian, Geok Chin Tan, Yin Ping Wong

**Affiliations:** 1 Department of Pathology, Faculty of Medicine, Universiti Kebangsaan Malaysia, Kuala Lumpur 56000, Malaysia; p109432@siswa.ukm.edu.my (B.I.A.W.); roslina.arshad@ukm.edu.my (R.M.A.); nurwardah@ppukm.ukm.edu.my (N.A.); 2 Department of Community Health, Faculty of Medicine, Universiti Kebangsaan Malaysia, Kuala Lumpur 56000, Malaysia; drsham@hctm.ukm.edu.my

**Keywords:** biomarker, endometrial carcinoma, EZH2, immune checkpoint, immunohistochemistry, PD-L1, prognosis, survival

## Abstract

**Background:** This study investigated PD-L1 and EZH2 immunoexpressions in endometrial carcinomas (ECs) and correlated their associations with clinicopathological parameters and five-year survival outcomes. **Methods:** A cross-sectional, retrospective study was conducted on all ECs diagnosed between January 2014 and December 2018. Immunohistochemical staining for PD-L1 (clone 22C3) and EZH2 was performed on tumour samples, and their expression levels were assessed. **Results:** Among the 104 EC cases included, 19.2% (*n* = 20) overexpressed PD-L1, while 8.7% (*n* = 9) overexpressed EZH2. Most (*n* = 19/20, 95.0%) PD-L1-expressing tumour cells showed EZH2 immunonegativity. Likewise, most (*n* = 8/9, 88.9%) EZH2-expressing ECs were PD-L1-negative. Increased PD-L1 and EZH2 expressions in ECs were seen more frequently in women more than 60 years of age (*p* = 0.013 and *p* = 0.039). EZH2 overexpression was associated with higher tumour grade (*p* = 0.009) and more aggressive histological subtypes (*p* = 0.013), while PD-L1 expression was not significantly associated with tumour grade, tumour stage, histological subtypes, and lymph node status (*p* > 0.05). Kaplan–Meier survival analysis revealed that PD-L1-positive ECs had a significantly better five-year overall survival (OS) rate compared to PD-L1-negative ECs (*p* = 0.034). Conversely, EZH2 overexpression did not correlate with survival outcomes (*p* > 0.05). Notably, the combination of PD-L1 and EZH2 expression patterns on ECs (PD-L1-/EZH2+) portends the worst OS compared to other combined PD-L1/EZH2 expression patterns (*p* = 0.05). **Conclusions:** PD-L1 immunoexpression was associated with better survival outcomes in ECs, while overexpression of EZH2 was associated with higher tumour grade and aggressive histological subtypes, suggesting their potential utility as prognostic biomarkers in ECs.

## 1. Introduction

The incidence of endometrial carcinomas (ECs) is on the rise globally, with low- and middle-income countries facing greater challenges due to low public awareness and limited access to screening programmes [1]. According to the World Cancer Research Fund International, ECs are the 15th most common cancer among women in 2022, with approximately 420,368 new reported cases worldwide. In Malaysia, data extracted from the National Cancer Registry (2012–2016) revealed an incidence rate of 9.4 cases per 100,000 women [2].

There are several factors that influence the prognosis of ECs, including tumour grade, histological subtype, depth of myometrial invasion, and the extent of cervical and adnexal involvement. Recent advances in molecular classification by The Cancer Genome Atlas (TCGA) Research Network have refined risk stratification of ECs, categorising them into four key molecular subtypes that can be identified by surrogate immunomarkers: POLE-mutated, mismatch repair (MMR)-deficient, p53-abnormal, and no specific molecular profile (NSMP) [3]. POLE-mutated ECs typically exhibit a favourable prognosis, while p53-abnormal tumours are associated with a poor prognosis [3].

Immune system evasion is one of the hallmarks of cancer biology, allowing cancer cells to evade immune surveillance. The programmed cell death protein-1 (PD-1) and its ligand, programmed cell death ligand-1 (PD-L1), play a critical role in immune modulation. By tampering with this pathway, the cancer cells can evade detection and suppress the body’s immune response. PD-1, a receptor found on the surface of tumour-specific activated T cells, B lymphocytes, and natural killer cells, interacts with its ligand, programmed cell death ligand-1 (PD-L1)—a transmembrane protein expressed by cancer cells. When PD-L1 binds to PD-1, it suppresses the T-cell response, thereby facilitating immune evasion and promoting tumour progression [4]. Interestingly, the prognostic significance of PD-L1 expression varies across different cancer types. While PD-L1 immunoreactivity has been associated with improved outcomes in colorectal and lung cancers [5,6], it correlates with reduced survival in gastric cancer [7], hepatocellular carcinoma [8], bladder cancer [9], and oesophageal carcinoma [10]. In ECs, the prognostic relevance of PD-L1 remains elusive. Lu et al. (2020) reported that PD-L1 overexpression had little to no significant prognostic impact on overall survival (OS) [11], while other studies have shown conflicting results [12].

The discovery of immune checkpoint inhibitors, such as PD-1/PD-L1 inhibitors, has revolutionised treatment strategies for patients with cancer, thereby significantly enhancing clinical outcomes and OS. The U.S. Food and Drug Administration (FDA) recently approved anti-PD-1 therapy—Pembrolizumab (marketed as Keytruda) for the treatment of advanced and recurrent ECs [13]. This highlights the growing need for a better understanding of PD-L1 immunoexpression in ECs, particularly in identifying patients who may potentially benefit from this targeted immunotherapy.

Enhancer of zeste homolog 2 (EZH2) is an essential molecular biomarker in oncology, playing a critical role in epigenetic gene regulation. As the catalytic subunit of the polycomb repressive complex 2 (PRC2), EZH2 mediates tri-methylation of histone H3 at lysine 27 (H3K27me3), resulting in transcriptional repression of tumour-suppressor genes and other regulatory elements [14]. Through modulation of gene expression, EZH2 influences various cellular processes, including promoting cell survival, proliferation, epithelial-to-mesenchymal transition, invasion, and drug resistance [15].

EZH2 is frequently dysregulated in many cancer cell types, contributing to uncontrolled cell proliferation and tumourigenesis [16,17]. Elevated EZH2 expression is often associated with aggressive tumour behaviour and poor prognostic outcomes in a myriad of malignancies, including ECs [18,19]. For instance, a meta-analysis by Fan et al. (2020), involving 2180 cases of non-small cell lung carcinoma (NSCLC), revealed that high EZH2 expression in lung adenocarcinoma indicated a poorer prognosis with shorter median survival time [20]. Similarly, another meta-analysis included 1049 gliomas and reported that EZH2 positivity was significantly correlated with worse OS and progression-free survival (PFS) [21].

More recently, EZH2 has gained significant attention for its potential as a therapeutic target for cancer therapies, particularly in lung cancers. Zhang and colleagues successfully developed EZH2 inhibitor JQEZ5 and demonstrated its antitumour effects in EZH2-driven lung adenocarcinoma models [22]. Additionally, Wang et al. (2022) showed that inhibition of EZH2 signalling ameliorated the proliferation rate of NSCLC cells via downregulation of the PD-L1 pathway [23]. The relationship between EZH2 and PD-L1 expression in various malignancies, including ECs, however, requires further investigation.

In the present study, we aimed to assess the immunoexpressions of PD-L1 and EZH2 in ECs and evaluate their associations with key clinicopathological parameters, including patients’ age, tumour grade and stage, depth of myometrial invasion, histological subtype, and survival outcomes.

## 2. Materials and Methods 

### 2.1. Study Design and Subjects

This was a single-centre, cross-sectional retrospective study conducted at a tertiary referral centre, Hospital Canselor Tuanku Muhriz (HCTM), involving archival formalin-fixed paraffin-embedded (FFPE) tissue blocks from histologically confirmed EC cases between January 2014 and December 2018.

We included all cases of histologically confirmed ECs involving patients who had undergone total hysterectomy and had a minimum follow-up period of five years post-surgery. Overall survival (OS) was defined as the time elapsed between the date of definitive surgery and the date of last follow-up or death from any cause. Exclusion criteria included cases with missing tissue blocks, tissue biopsies-only specimens, histological subtypes other than carcinomas, those lacking sufficient clinicopathological data, or patients who defaulted on follow-up.

Relevant clinicopathological data, such as patients’ age, ethnicity, depth of myometrial invasion, TNM stage, FIGO stage, lymph node status at presentation, and five-year survival status, were retrieved from the Integrated Laboratory Management System (ILMS) and patient medical records. All data were anonymous, and each subject was assigned a coded identifier.

### 2.2. Immunohistochemistry Staining Method

The tissue blocks were sectioned at approximately 4 µm thickness and mounted on adhesive glass slides (Platinum Pro White, Matsunami, Japan). The slides were air-dried at room temperature overnight before being incubated on a hot plate at 60 °C for 1 h.

Immunohistochemical staining was carried out on the tissue sections using the EnVision^TM^ FLEX Mini Kit (Code No. K8023, Dako, Hovedstaden, Denmark). Primary antibodies were diluted to optimal concentration using Dako REAL^TM^ Antibody Diluent (Code No. S2022, Dako, Hovedstaden, Denmark) (Table 1). Washing steps were performed using EnVision^TM^ FLEX Wash Buffer 20X (Code No. K8007, Dako, Hovedstaden, Denmark) and diluted with deionised water. DAB-containing substrate solution was prepared by mixing 50× concentrated EnVision^TM^ FLEX DAB+ Chromogen with EnVision^TM^ FLEX^TM^ Substrate Buffer (Code No. K8023, Dako, Hovedstaden, Denmark). Deparaffinisation and pre-treatment steps were performed in a Decloaking Chamber™ NxGen (Ref. No: DC2012–220V, Biocare Medical, Pacheco, CA, USA) at 110 °C for 30 min, followed by 30 min of cooling. The slides were rinsed with running tap water for 3 min, incubated with EnVision^TM^ FLEX Peroxidase-Blocking Reagent (Code No. DM821, Dako, Hovedstaden, Denmark) for 10 min, and followed by a washing step.

The slides were first incubated at room temperature with the primary antibody, followed by incubation with EnVision^TM^ FLEX Mouse Linker (Code No. K8012/K8022, Dako, Hovedstaden, Denmark). Next, they were incubated with secondary antibody EnVision^TM^ FLEX HRP (Code No. K8023, Dako, Hovedstaden, Denmark) for 30 min. The sections were then treated with DAB-containing substrate working solution for 7 min. After completion of these steps, the slides were counterstained with Haematoxylin 2 (ThermoScientific, Waltham, MA, USA), dehydrated through increasing concentrations of alcohol (80%, 90%, 100%, and 100%), and cleared with xylene. Finally, the slides were mounted using CoverSeal^TM^-X xylene-based mounting medium (Cat. No.: FX2176, Cancer Diagnostics, Durham, NC, USA).

The immunohistochemistry staining results were independently evaluated and scored by three histopathologists—one junior (B.I.A.W.) and two experienced histopathologists (Y.P.W. and G.C.T.), who were blinded to histological diagnosis and patient outcomes. Discrepancies were resolved by consensus using a multi-headed microscope.

### 2.3. Evaluation of PD-L1 Immunohistochemical Stain

PD-L1 immunoreactivity was defined as partial or complete brown membrane staining of tumour or immune cells at any intensity. The combined positive score (CPS) was calculated as the number of PD-L1-positive tumour cells and immune cells (lymphocytes and macrophages) divided by the total number of tumour cells. A CPS greater than 1 was considered positive for PD-L1 immunoexpression [24].

### 2.4. Evaluation of EZH2 Immunohistochemical Stain

EZH2 immunoreactivity was determined by the presence of brown nuclear staining in the cells of interest. The percentage of positive cells was scored as follows: 0–30% (1 point), 31–70% (2 points), and 71–100% (3 points). Staining intensity was graded as follows: no staining (0 point), light yellow (1 point), brown (2 points), and strong brown (3 points). The total score was the product of the percentage and intensity scores, with a score of 3 or higher considered positive [25].

### 2.5. Statistical Analysis

The statistical analysis was performed using SPSS 26.0 software (IBM, Armonk, New York, USA). Demographic and clinicopathological characteristics between PD-L1-expressed, EZH2-expressed, and non-expressed ECs were compared using the Chi-square test, student *t*-test, and Fisher’s exact test. Survival analysis was conducted using the Kaplan–Meier method. The five-year OS curves were compared using the log-rank test. A *p*-value of less than 0.05 was regarded as statistically significant.

## 3. Results

### 3.1. Frequency and Demographic

A total of 104 histologically diagnosed ECs met the inclusion and exclusion criteria. In this cohort, most patients were aged 60 years or older (54.8%, *n* = 57/104), with a mean age of 59.10 ± 10.6 years. The majority of the patients were Malay (55.8%, *n* = 58/104), followed by Chinese (33.6%, *n* = 35/104), Indian (8.7%, *n* = 9/104), and other ethnicities (1.9%, *n* = 2/104). Endometrioid carcinoma was the predominant histological subtype (76.9%, *n* = 80/104), followed by serous carcinoma (12.5%, *n* = 13/104), clear cell carcinoma (3.8%, *n* = 4/104), mucinous carcinoma (1.9%, *n* = 2/104), and other subtypes (4.8%, *n* = 5/104). Regarding tumour grade, 57.7% (*n* = 60/104) were FIGO grade 1, 18.3% (*n* = 19/104) were grade 2, and 24.0% (*n* = 25/104) were FIGO grade 3. Based on pathological T staging, most tumours were pT1 (67.3%, *n* = 70/104), followed by pT3 (19.2%, *n* = 20/104), pT2 (12.5%, *n* = 13/104), and pT4 (0.9%, *n* = 1/104). As for FIGO staging, the majority were in FIGO stage I (60.6%, *n* = 63/104), followed by FIGO stage III (21.1%, *n* = 22/104), FIGO stage II (12.5%, *n* = 13/104), and FIGO stage IV (5.8%, *n* = 6/104). More than half (51.9%, *n* = 54/104) of the tumours were limited to the endometrium or involved less than 50% of the myometrium. Of the 71 patients who had pelvic lymph nodes removed during radical hysterectomy, 80.3% (*n* = 57/71) had no lymph node metastases at diagnosis, while 19.7% (*n* = 14/71) individuals had confirmed lymph node involvement at presentation (Table 2).

### 3.2. PD-L1 Immunoexpression and Its Correlation with Clinicopathological Parameters

**Figure 1:**
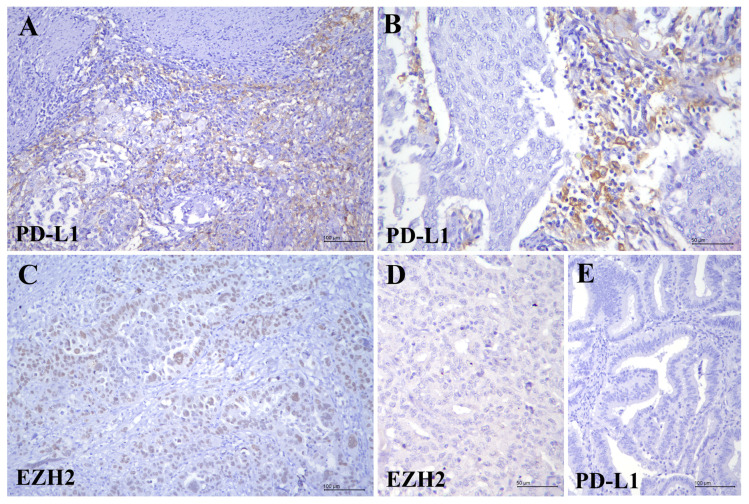
PD-L1 and EZH2 in endometrial carcinoma. (**A**) Both tumour cells and tumour-infiltrating lymphocytes show PD-L1 immunopositivity (PD-L1, ×200); (**B**) PD-L1 positivity restricted to tumour-infiltrating lymphocytes (PD-L1, ×400). (**C**) Tumour cells show EZH2 immunopositivity (EZH2, ×200). The tumour cells are negative for (**D**) EZH2 (EZH2, ×400) and (**E**) PD-L1 (PD-L1, ×200).

We observed that 19.2% (*n* = 20/104) of EC cases were positive for PD-L1, while the remaining 80.8% (*n* = 84/104) were PD-L1-negative (Table 2, Figure 1). Among patients aged 60 or older, 28.1% (*n* = 16/57) were PD-L1-positive, compared to 8.5% (*n* = 4/47) among younger patients, and the difference was statistically significant (*p* = 0.013). There was no significant correlation between PD-L1 immunoexpression and ethnicity, histological subtype, FIGO grade, pathological T stage, FIGO stage, depth of myometrial invasion, or lymph node metastasis (*p* > 0.05) (Table 2). Notably, we observed that 95.0% (*n* = 19/20) of PD-L1 expressing tumour cells were negative for EZH2, although this inverse relationship was not statistically significant (*p* > 0.05) (Table 3).

### 3.3. PD-L1 Immunoexpression with Five-Year Overall Survival

**Figure 2:**
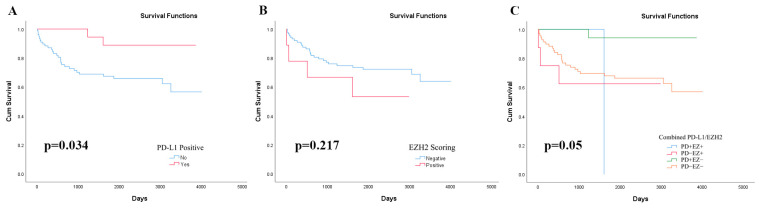
Kaplan–Meier survival plot for overall survival (OS) in patients with endometrial carcinoma. (**A**) OS in PD-L1-positive group and PD-L1-negative group; (**B**) OS in EZH2-positive group and EZH2-negative group; and (**C**) OS in various combined PD-L1 and EZH2 immunoexpression patterns.

Of the 104 patients diagnosed with ECs, 67 (64.4%) remained alive and disease-free, 27 (26.0%) had died from advanced or recurrent ECs, 7 (6.7%) were lost to follow-up, and 3 (2.9%) died of non-cancer-related causes. Of these patients who were still alive and well, 18 (26.9%) showed PD-L1 immunoexpression. Kaplan–Meier survival analysis revealed significantly better five-year OS in PD-L1-positive patients compared to PD-L1-negative ones (*p* = 0.034) (Figure 2).

### 3.4. EZH2 Immunoexpression and Its Correlations with Clinicopathological Parameters

EZH2 immunoexpression was rare in ECs, with only 8.7% (*n* = 9/104) of the cases testing positive, while the remaining 91.3% (*n* = 95/104) were negative for EZH2 (Table 2, Figure 1). EZH2 immunopositivity showed a statistically significant correlation with higher tumour grade (*p* = 0.009) and more aggressive histological subtypes such as serous carcinoma (*p* = 0.013). It was also observed more frequently in patients aged 60 and above (*p* = 0.039). No significant association was found between EZH2 expression and other clinicopathological parameters, including ethnicity, pathological T stage, FIGO stage, depth of myometrial invasion, or lymph node metastasis (*p* > 0.05) (Table 2). Additionally, 88.9% (*n* = 8/9) of EZH2-expressing ECs were PD-L1-negative, though not statistically significant (Table 3).

### 3.5. EZH2 Immunoexpression with Five-Year Overall Survival

Of the 67 patients who remained alive and disease-free, 5 (7.5%) showed EZH2 immunoexpression. Kaplan–Meier survival analysis revealed that patients with EZH2 immunoexpression had poorer five-year OS compared to those without, although the difference was not proven to be statistically significant (*p* > 0.05) (Figure 2).

### 3.6. Combined PD-L1 and EZH2 Immunoexpressions with Five-Year Overall Survival

Of the 67 patients who remained alive and disease-free, the majority (*n* = 44/67, 65.7%) expressed neither PD-L1 nor EZH2, while 18 (26.8%) were PD-L1+/EZH2-, and 5 (7.5%) were PD-L1-/EZH2+. No cases co-expressed both PD-L1 and EZH2. Kaplan–Meier survival curves revealed that the PD-L1-/EZH2+ group portends a significantly poorer five-year OS compared to the other expression profiles (*p* = 0.05) (Figure 2).

## 4. Discussion

Endometrial carcinoma is the sixth most common malignancy among women in Malaysia, with an incidence rate of 4.7% between 2012 and 2016 [2]. In our study, we analysed 104 cases of ECs, with endometrioid carcinoma being the predominant histological subtype (*n* = 80/104, 76.9%), consistent with findings from previous studies [24,26].

A number of studies have previously investigated the expression of PD-L1 in ECs [24,26,27,28], yielding inconsistent results. In the present study, the incidence of PD-L1 expression was 19.2% (*n* = 20), aligning with prior reports that ranged between 17% and 62% [26,27,28]. Variability in PD-L1 immunoexpression may be attributed to differences in antibody clones, and the composition of the studied population and scoring criteria, hence emphasising the need for standardised antibodies and the staining interpretation methods.

Notably, PD-L1 immunoexpression in ECs showed a significant association with the older age group, in agreement with that of earlier studies [26,29]. Recently, Onorati et al. (2022) reported that PD-L1 was upregulated in naturally senescent cells via the JAK–STAT pathway, contributing to immune cell inactivation as part of the aging process [30]. Upregulation of PD-L1 expression in senescent cells may contribute to an overall immunosuppressive environment that impairs immune-mediated clearance of damaged cells, which drives tumourigenesis. This could explain the high incidence of cancer in elderly individuals [30].

The role of PD-L1 as a prognostic biomarker in ECs remains somewhat controversial. While accumulating evidence suggests that PD-L1 immunoexpression is associated with advanced tumour stage, higher tumour grade, aggressive histological subtypes, and lymph node metastasis [11,24,26], some other studies reported otherwise [12]. The present study concluded that PD-L1 immunoexpression was independent of clinicopathological parameters such as ethnicity, histological subtype, pathological T stage, or lymph node metastasis at presentation. Nevertheless, we observed higher PD-L1 expression in clear cell carcinoma (50%) and in patients without lymph node involvement (18.9%). These findings were in line with those of Ghasemi et al. (2022), who found a significant association between PD-L1 expression and clear cell morphology, especially in the endometrium [31].

While some ongoing studies reported a link between high PD-L1 immunoexpression and poor survival outcomes [24,26,32], the present study suggested the opposite—PD-L1 positivity was associated with significantly better five-year survival outcomes. Our findings concurred with the studies of Chen et al. (2022) and Zong et al. (2021), both of which reported improved survival outcomes among PD-L1-positive EC patients [33,34]. We hypothesised that PD-L1 immunoexpression may reflect an active antitumour immune response and an immune-activated tumour microenvironment. Another explanation for this observation is that since the ECs that we included in our study were predominantly low-grade tumours and of endometrioid histological subtype, we proposed that in certain histological subtypes of ECs where PD-L1 is expressed, it is associated with a more favourable prognosis. In other words, the tumour microenvironment may not be the only factor that plays a significant role in determining the prognostic implications of PD-L1 expression in ECs.

In the present study, EZH2 expression was rare, detected in only 8.7% of the cases. This is in contrast to earlier reports, where EZH2 immunoexpression was observed in up to 63.6% [35] and 68.3% [25] of ECs. Similar to PD-L1, EZH2 immunoexpression was significantly correlated with older age. Our findings contrasted with other studies, which found no correlation between EZH2 expression and the menopausal status or patient age [25,36].

EZH2 has been implicated in the pathogenesis and progression of several cancers, where its overexpression often correlates with aggressive tumour behaviour and poorer prognosis, in agreement with the current study. Gu et al. (2017) showed that EZH2 overexpression in ECs was associated with deeper myometrial invasion, higher tumour grade, lymph node metastasis, and advanced TNM stage [25]. This could be related to the proliferative and invasive abilities of EZH2-expressing malignant cells. Gu et al. (2017) also demonstrated that knockdown of EZH2 significantly reduced the invasive ability of EC RL-952 cells in vitro [25].

With regards to survival, Oki et al. (2017) revealed that overexpression of EZH2 in ECs was associated with a decreased OS compared to EZH2-negative ECs [37]. Similarly, a study by Roh et al. (2020) showed that EZH2 overexpression was significantly correlated with decreased PFS and OS in ECs [36]. In contrast, in our study cohort, ECs that overexpressed EZH2 did not seem to be significantly associated with a lower five-year OS. The lack of significant clinical correlations in this study may be due to the limited sample size or population differences, or it might reflect a genuine lack of association between EZH2 expression and survival in ECs, requiring larger, multi-centre studies.

Noteworthy, PD-L1 expression was found to be negatively correlated with EZH2 expression in ECs. Our findings aligned with a mechanistic study by Xiao et al. (2019) in hepatocellular carcinoma (HCC), which demonstrated a similar negative correlation between EZH2 and PD-L1 expression [38]. The authors proposed that EZH2 suppresses PD-L1 expression via epigenetic modification, by upregulating H3K27me3 levels at the *CD274* (which encodes PD-L1) and *IRF1* gene in hepatoma cells, highlighting the critical role of EZH2 in reshaping the tumour immune microenvironment [38].

We conducted the first survival analysis using the combined expression patterns of PD-L1 and EZH2 in ECs. The current study demonstrated that the combination expression pattern of PD-L1 and EZH2 in ECs was associated with patients’ prognosis, with PD-L1-/EZH2+ expression profile correlating with poorer five-year OS. While high levels of EZH2 expression were associated with poor OS and relapse-free survival (RFS) in patients with HCC, intriguingly, Xiao et al. (2019) reported that low EZH2-expressing HCC that co-expressed PD-L1 (PD-L1+/EZH2^low^) was linked to poorer OS and RFS [38]. This association, however, was not observed in cases with high EZH2 expression (PD-L1+/EZH2^high^) or other combined PD-L1/EZH2 expression patterns [38], highlighting the complexity of their interaction.

In the current molecular classification of ECs, PD-L1 expression is significantly more pronounced in POLE-mutated and MMR-deficient subtypes, both of which are associated with more favourable clinical outcomes compared to the p53-abnormal and NSMP subgroups [34,39]. These studies demonstrated that PD-L1 was expressed not only in the tumour cells but also in tumour-infiltrating lymphocytes. Moreover, PD-L1 positivity has been shown to correlate with increased T-cell infiltration, especially CD3+ and CD8+ lymphocytes [12]. The relationship between EZH2 expression and the TCGA molecular subgroups, however, has not been previously reported.

The observed expression patterns of PD-L1 and EZH2 in ECs have raised potential therapeutic considerations. PD-L1 positivity, linked to improved five-year OS in our cohort, identifies a subset of patients with EC who could benefit from PD-1/PD-L1 immune checkpoint inhibitors (ICIs) such as pembrolizumab or dostarlimab [12,13]. Tumours exhibiting PD-L1 expression have shown heightened responsiveness to ICIs, supporting the use of immunotherapy as a promising treatment option, particularly in advanced or recurrent disease settings [12,13]. EZH2, which promotes carcinogenesis and is highly expressed in a myriad of human cancers, serves as a perfect target for cancer therapy. Tazemetostat, the first FDA-approved EZH2 inhibitor, has demonstrated significant clinical benefits in treating epithelioid sarcoma and follicular lymphoma [40]. The potential application of EZH2 inhibitors in ECs, however, remains to be further explored.

The present study has several limitations. While the sample size was adequate for exploratory analysis, it limited subgroup comparisons based on clinicopathological parameters. Additionally, the retrospective design of the present study and reliance on archival tissues may introduce biases related to case selection and tissue preservation.

## 5. Conclusions

PD-L1 immunoexpression was associated with better survival outcomes in ECs, while EZH2 overexpression was correlated with higher tumour grade and aggressive histological subtypes. The inverse relationship between these biomarkers suggests their distinct biological roles and potential utility as prognostic biomarkers. Future large-scale, prospective studies are warranted to validate these results and explore the therapeutic implications of targeting PD-L1 and EZH2 in the management of ECs.

## Figures and Tables

**Table 1:** Immunohistochemical antibodies.

Primary Antibody	Product Code/Source	Dilution
PD-L1, clone 22C3, mouse monoclonal	M365329, Dako, Denmark	1:50
EZH2, mouse monoclonal	415M-14, Cell Marque, USA	1:100

**Table 2:** Correlations of PD-L1 and EZH2 immunoexpressions in endometrial carcinomas with clinicopathological parameters.

Clinical Characteristic		Total *n* = 104 (%)	PD-L1 + ve *n* = 20 (%)	PD-L1 − ve *n* = 84 (%)	*p* Value	EZH2 + ve *n* = 9 (%)	EZH2 − ve *n* = 95 (%)	*p* Value
Age at diagnosis	<60 years ≥60 years	47 (45.2) 57 (54.8)	4 (20.0) 16 (80.0)	43 (51.2) 41 (48.8)	0.013 *	1 (11.1) 8 (88.9)	46 (48.4) 49 (51.6)	0.039 *
Ethnic	Malay Chinese Indian Others	58 (55.8) 35 (33.7) 9 (8.7) 2 (1.9)	7 (35.0) 12 (60.0) 1 (5.0) 0 (0.0)	51 (60.7) 23 (27.4) 8 (9.5) 2 (2.4)	0.063	5 (55.6) 3 (33.3) 1 (11.1) 0 (0.0)	53 (55.8) 32 (33.7) 8 (8.4) 2 (2.1)	1.000
Histological subtypes	Endometrioid	80 (76.9)	16 (80.0)	64 (76.2)	0.362	3 (33.3)	77 (81.1)	0.013 *
	Serous	13 (12.5)	1 (5.0)	12 (14.3)		5 (55.6)	8 (8.4)	
	Clear cell	4 (3.8)	2 (10.0)	2 (2.4)		0 (0.0)	4 (4.2)	
	Mucinous	2 (1.9)	0 (0.0)	2 (2.4)		0 (0.0)	2 (2.1)	
	Others	5 (4.8)	1 (5.0)	4 (4.8)		1 (11.1)	4 (4.2)	
FIGO grade	1	60 (57.7)	11 (55.0)	49 (58.3)	0.698	2 (22.2)	58 (61.1)	0.009 *
	2	19 (18.3)	5 (25.0)	14 (16.7)		1 (11.1)	18 (18.9)	
	3	25 (24.0)	4 (20.0)	21 (25.0)		6 (66.7)	19 (20.0)	
Pathological T stage	pT1	70 (67.3)	17 (85.0)	53 (63.1)	0.319	5 (55.6)	65 (68.4)	0.629
	pT2	13 (12.5)	2 (10.0)	11 (13.1)		1 (11.1)	12 (12.6)	
	pT3	20 (19.2)	1 (5.0)	19 (22.6)		3 (33.3)	17 (17.9)	
	pT4	1 (1.0)	0 (0.0)	1 (1.2)		0 (0.0)	1 (1.1)	
FIGO stage	I	63 (60.6)	14 (70.0)	49 (58.3)	0.621	5 (55.6)	58 (61.1)	1.000
	II	14 (13.5)	2 (10.0)	12 (14.3)		1 (11.1)	13 (13.7)	
	III	21 (20.2)	4 (20.0)	17 (20.2)		2 (22.2)	19 (20.0)	
	IV	6 (5.8)	0 (0.0)	6 (7.1)		1 (11.1)	5 (5.3)	
Depth of myometrial invasion	<50%	54 (51.9)	12 (60.0)	42 (50.0)	0.464	4 (44.4)	50 (52.6)	0.735
	≥50%	50 (48.1)	8 (40.0)	42 (50.0)		5 (55.6)	45 (47.4)	
#Lymph node metastasis	Yes	14 (19.7)	2 (12.5)	12 (21.8)	0.501	3 (42.9)	11 (17.2)	0.133
	No	57 (80.3)	14 (87.5)	43 (78.2)		4 (57.1)	53 (82.8)	

* statistically significant; # missing data.

**Table 3:** Correlation between PD-L1 and EZH2 immunoexpressions in ECs.

	Total *n* = 104	PD-L1 +ve *n* = 20 (%)	PD-L1 −ve *n* = 84 (%)	*p* Value
**EZH2 + ve**	9 (8.7)	1 (5.0)	8 (9.5)	0.687
**EZH2 − ve**	95 (91.3)	19 (95.0)	76 (90.5)	

+ve—positive; −ve—negative.

## Data Availability

No new data were created or analyzed in this study.

## Abbreviations

The following abbreviations are used in this manuscript:

EC	Endometrial carcinoma
EZH2	Enhancer of zeste homolog 2
FDA	Food and Drug Administration
FFPE	Formalin-fixed paraffin-embedded
HCCHCTM	Hepatocellular carcinomaHospital Canselor Tuanku Muhriz
MMR	Mismatch repair
NSCLC	Non-small cell lung carcinoma
OS	Overall survival
PD-1	Programmed cell death-1
PD-L1	Programmed cell death ligand-1
PRC2	Polycomb repressive complex 2
PFS	Progression-free survival
RFS	Relapse-free survival
